# Supplementary material for: Impact of trainability on telomere dynamics of pet dogs (Canis lupus familiaris): An explorative study in aging dogs
Source: PLoS One. 2025 Feb 5;20(2):e0317332. doi: 10.1371/journal.pone.0317332 (PMC11798472; doi:10.1371/journal.pone.0317332)
Supplement: S2 Table — (PDF) [file pone.0317332.s002.pdf]

Table S2. Dog breeds grouped into FCI groups based on the Fédération Cynologique Internationale (FCI) website (https://www.fci.be/en/).

FCI group name in this study	FCI groups based on the Fédération Cynologique Internationale homepage	Dog breeds in the FCI group based on the Fédération Cynologique Internationale homepage
mix (25 individuals)	-	mixed breeds
gr. 1 (19 individuals)	1	sheep dogs and cattle dogs
gr. 8 (6 individuals)	8	retrievers, flushing - and water dogs
diff (13 individuals)	2<br>3<br>4<br>5<br>6<br>7<br>9	swiss mountain dogs<br>terriers<br>dachshunds<br>spitz and primitive types<br>scent hounds<br>pointing dogs<br>companion and toy dogs
