# Supplementary material for: Impact of trainability on telomere dynamics of pet dogs (Canis lupus familiaris): An explorative study in aging dogs
Source: PLoS One. 2025 Feb 5;20(2):e0317332. doi: 10.1371/journal.pone.0317332 (PMC11798472; doi:10.1371/journal.pone.0317332)
Supplement: Attachment — Submitted filename: Response to Reviewers.docx [file pone.0317332.s003.docx]

5. Review Comments to the Author

Please use the space provided to explain your answers to the questions above. You may also include additional comments for the author, including concerns about dual publication, research ethics, or publication ethics. (Please upload your review as an attachment if it exceeds 20,000 characters)

Answer:
Dear Reviewers,

Thank you for your thorough review of our manuscript. We greatly appreciate the time and effort you have dedicated to evaluating this study and for providing insightful comments and suggestions.
In the revised manuscript, we have addressed each of your comments in detail. Please find our point-by-point responses below. We hope the revisions meet your expectations and enhance the quality of our work. Thank you again for your valuable feedback.

Best regards.

Reviewer #1: This is a comparison of percentage telomere length change after approximately one year of two dietary treatments in dogs. I like the topic and appreciate the effort made. I think there is value in these data. I am not convinced of the appropriateness of the statistical analyses and interpretation. I encourage reconsideration of a revised manuscript that reports this work.

The major issues are 1) predictor description and quantification, and 2) the statistical analyses employed.

1) All evaluated predictors need to be explained in the Materials and Methods. At least the predictor ‘trainability’ was somewhat explained in the results section. I also note that trainability may be inherent in dogs (lines 299-300) or developed across time (line 292). If it is the latter, that may complicate the interpretation of results.

Answer: Thank you for this valuable comment. We included Table 1. in the methods section to explain all variables used for our modeling approach. The study by Durga et al. 2020 found that trainability does not change with age. We included a sentence in the discussion to explain this phenomenon line 308-313.

2) The statistical methodology used is pretty nonstandard. Why not just use analysis of variance that is interpretable by most researchers?

a. The modeled effects must be clarified and defined. For example, were the behavior assessments modeled as linear covariates or as categorical variables?—presentation of results suggests both. They are difficult to see, especially in Table 1. More description of the distributions of these values would be best.

Answer: We disagree with the reviewer, as the chosen statistics are standard methods in exploratory studies (e.g. Stefanović, M et al. 2021, <https://doi.org/10.1186/s12862-021-01856-z>; Hadinger et al, 2015, <https://doi.org/10.1111/2041-210X.12422>;Eom, Tae-Kyung, et al. 2018, https://doi.org/10.25225/fozo.v67.i3-4.a5.2018). We used a model averaging approach as we were not testing a specific hypothesis and therefore, we included all variables in one large model. The number of predictors would lead to massive overfitting and together with the number of models calculated interpretation of p-values would be incorrect. So instead of calculating p-values, we determined the relative variable importance of the predictores. This approach shows the most important variable as 1.0 (in our case “trainability”) and the next lower variable (in our case “age at first sampling”) has a value of 0.39. So “age at first sampling” is only 39% as important as “trainability”. If a variable is not included in the RVI-table it is not important at all for the models.

Concerning the linear and categorical use of the predictors, we included information about the character of the variables (e.g. numeric or categorical) in table 1.

We furthermore included more information on the symbols used in table 2.

b. AICc—I am not familiar with. Akaike’s information criterion (without the little c) is maybe useful for random structure determination but is less good for fixed effect determination. What would be wrong with just a simple model with evaluated effects?
Analysis software (MUMin) should be in the Methods section.

Answer: We used the Akaike information criterion - corrected for small sample sizes. We did not intend to determine any fixed effects but rather used it to determine the best models.
The Akaike Information Criterion (AIC) estimates prediction error and ranks statistical models by relative quality. A corrected version, AICc, performs better with small sample sizes but aligns with AIC for large samples. Models with a delta (ΔAICc) below 2 are distinctly different. AICc weights indicate the relative likelihood of a model (1.0 being the most likely), making them ideal for ranking models. Lower AIC or AICc values signify better model fit, while ΔAIC/AICc measures the difference between the best model and others.
As we did not test a hypothesis but used an exploratory approach, it would be incorrect to use a simple model with evaluated effects.

We agree that we have to mention the R-packages not only in the results section, but also in the methods. We therefore included it - line 229-230.

c. FCI group is never defined.

Answer: Thank you for raising this important point. We have addressed this by including more information on FCI groups in the methods section, see line 133-139. Furthermore we created table S2 that shows how the breeds in our study are arranged in FCI groups.

d. Table 1—define table elements and numbers. Does this table mean that age was only important in results from two models? This is pretty inconsistent with telomere length investigations in many species and should be explained.

Answer: Thank you for this comment. We explain the table elements in the heading of table 2 - line 290-291.
Age was indeed only included in two models that had a delta AICc under two. As stated in line 53-59 telomere dynamics are complex and do not necessarily show a negative trend with aging. We also included a paragraph about age and telomere dynamics in the discussion line 308-313.

e. Lines 234-235 all possible additive models—this could mean a large number of analyses giving the opportunity to find something meaningful. Multiple testing issues may apply.

Answer: We are aware that multiple testing could be a problem. But as we were not calculating p-values and did not interpret the models independently, multiple testing is not an issue. We have rewritten this part of the methods section to make this clearer - line 254-259.

f. Lines 236-237 relative variable importance is not familiar to me and may benefit from additional description.

Answer: Thank you for your advice. We included a paragraph about relative variable importance (RVI) in the methods section line 258-259.

g. Table 2 All Standard Errors are larger than the estimates except for trainability (high). The number of dogs supporting this estimate would be good to know (looks like 14 of the 63 from figure 2). In figure 2, it looks like one sample strongly influences this and another 4 may be above the average POTC. This figure clarifies that this variable was categorical rather than a covariate.

Answer: We appreciate your comment. As seen in Figure 2 thirteen dogs are part of the high trainability group. The detailed declaration of dogs per group and category can be found in Supplement Table 1. We did not include this information in detail in the text for all the groups to make the text as readable as possible. As already mentioned above, we included details about each variable (numeric or categorical) in Table 1. There are multiple samples in the trainability group medium and high that have a positive percentage of telomere change and we did not find any outliers in our analyses, so we are confident that there is no indication of single samples influencing the analyses.

h. lines 331-332 I think it is appropriate to be concerned about breed type since differential loss of telomeric sequences has been demonstrated for different breeds. Need to better describe the distribution of body weight in the text.

Answer: Thank you for your thoughtful comment.

We agree that telomeres differ in different breeds, however, in our study, we focused on social factors that were measured in all dogs the same way and did not correlate with the breed.

Following two publications describe telomere dynamics in different dog breeds (Benetos et al. 2011, <https://doi.org/10.1111/j.1474-9726.2011.00744.x>; Fick et al. 2012, <https://doi.org/10.1016/j.celrep.2012.11.021>) and find differences in dog breeds. But both papers used single-point measurements, while we measured telomeres repeatedly (with excellent reproducibility).

We also agree that it would have been great to use only purebred dogs for our study, but we were depending on volunteers. Unfortunately, a large fraction of dogs in our study were mixed breeds and therefore analysis on breed level was not feasible. This was the reason why we included FCI group instead of breed in the models.

Information of body weight (mean and range) are given in line 143-144. In addition, all data are available in Table S1.

3) Minor issues

a. Not a fan of first person plural for scientific papers. I acknowledge that it is personal opinion and not critical.

Answer: Thank you for sharing your perspective on the use of the first-person plural in scientific writing. We understand that preferences for writing style can vary, and we appreciate your acknowledgment that this is a personal opinion. While we aimed for clarity and engagement in our writing, we are always open to adapting our style to align with conventions and expectations. Your input is greatly valued, and we took it into consideration.

b. Some issues with English, such as incorrect usage of singular and plural nouns and verbs, capitalization, and awkward sentence construction.

Answer: Thank you so much for your detailed feedback! We truly appreciate you pointing out the issues with our English usage. We have carefully reviewed and revised the text to address errors in singular/plural forms, capitalization, and sentence construction. Your comments have been invaluable in helping us improve the clarity and precision of our writing, and we are committed to ensuring the highest linguistic quality in the revised version.

c. I recommend reporting results as past tense. Using present tense may imply that results are believed to accurately represent reality/truth. Scientific reports should probably be pretty conservative with respect to concluding truth.

Answer: Thank you for this suggestion! We agree that using past tense is more appropriate for reporting results, as it emphasizes the observations made during the study without implying absolute truths. We appreciate your insight and have revised our text accordingly to ensure it maintains the cautious and objective tone that is essential in scientific writing.

d. Out of sequence description of names, etc., (e.g., Clever Dog lab), decreases readability.

Answer: Thank you for this advice. Whenever possible, we made changes in the text, e.g. in line 101-105.

e. Names of veterinarians and data collectors should be omitted from the text.

Answer: Thank you for pointing this out! Following your suggestion, we have removed the names of veterinarians and data collectors from the text to ensure it aligns with standard practices. They now are only mentioned in the acknowledgements.

Reviewer #2: The article shows that repeated measurement of telomere length of cheek epithelial cells does not always show a decrease in length, and in some cases the length increases. Among many parameters studied, changes in telomere length are most strongly related to trainability. This is not a very well understood parameter. It is quickness of reaction, good appetite, cheerful disposition, increased motor activity, desire to play.

The disadvantages of the article include the following:

1. I have not found anywhere (except for the previous paper) an indication of the time between the first and second measurements.

Answer: Thank you very much for this valuable comment. We included the variable “time-between-sampling [weeks] in the table S1. In addition, we include the names of the dogs to make it easier for the readers to compare our data with the data of Durga et al. 2020.

2. There were 63 dogs involved in the study, but Fig. 1 shows only 16 points, Fig. 2 shows about 50 points. The authors should show the complete data.

Answer: We carefully checked our figures and Fig.2 shows all 63 data points (dogs). Fig.1 includes only 16 dots because this was the reproducibility test. We repeated one run with 16 samples to show the very good reproducibility of our telomere assay. This calculation was done in addition to the intraclass correlation coefficient (ICC) calculations. See also line 260 - 263.

3. Fig.2 shows 13 dots in the trainability level 3 group, but the original table shows only 12 dogs in this group. One is redundant.

Answer: Thank you for your thorough review of our manuscript. We carefully checked the table and counted 13 dogs in the trainability group 3.

4. I think the authors should show more raw data: it is very interesting, and most importantly it makes it easier to understand. It turns out that dogs have very different telomeres.

Answer: Table S1 contains all data that were used for this study. We included the names of the dogs in Table S1, so readers can easily compare it to the data in Durga et al. 2020.

5. It is necessary to describe the essence of the term trainability in more detail. Perhaps the authors should discuss the anomalous behavior of this parameter from the first article. It is the only one that does not decrease with age! This is strange. And the result of this study may have something to do with it.

Answer: Thank you for this comment. We include table 1 to the methods part that explains trainability (and all other variables used in the models) in more detail. We also included a possible explanation for the strange phenomenon that trainability does not change with age (line 308-313).

6. Classifying dogs based on questionnaires has a huge subjective component. Certainly, grasping the results of this paper, it should be repeated in a more objective way. It would be quite nice to measure blood cells in parallel.

I believe that the article will be worthy of publication in the Plos one journal after revision according to the remarks.

Answer: We agree that data based on questionnaires could cause problems concerning objectivity. In our dataset all behavioral variables (problem_solving, sociability, trainability, boldness, activity-independence, dependency) were measured in the Modified Vienna Canine Cognitive Battery (MVCCB) where all experiments were highly standardized and conducted the same way (for details see Durga et al. 2020). We included table 1 to explain all used variables of our models in more detail.

We also agree that it would be very interesting to analyze the blood cells (leukocytes from the buffy coat) at the same time. In the study described, we decided to use mucosal cells because they are non-invasive and are independent of health status at sample time point. See also line 173-175.

6. PLOS authors have the option to publish the peer review history of their article (what does this mean?). If published, this will include your full peer review and any attached files.

If you choose “no”, your identity will remain anonymous but your review may still be made public.

Do you want your identity to be public for this peer review? For information about this choice, including consent withdrawal, please see our Privacy Policy.

Reviewer #1: No

Reviewer #2: Yes: Dr. Yegor Yegorov
